# Molecular Cloning, Tissue Distribution and Antiviral Immune Response of Duck Src

**DOI:** 10.3390/genes15081044

**Published:** 2024-08-08

**Authors:** Jinlu Liu, Shuwen Luo, Guoyao Wang, Xuming Hu, Guohong Chen, Qi Xu

**Affiliations:** Key Laboratory for Evaluation and Utilization of Poultry Genetic Resources of Ministry of Agriculture and Rural Affairs, Yangzhou University, Yangzhou 225009, China; ljl15949082457@163.com (J.L.); santifay@163.com (S.L.); hxm@yzu.edu.cn (X.H.); ghchen2019@yzu.edu.cn (G.C.)

**Keywords:** duck, Src, IFN-β, innate immune

## Abstract

As a founding member of the Src family of kinases, Src has been confirmed to participate in the regulation of immune responses, integrin signaling, and motility. Ducks are usually asymptomatic carriers of RNA viruses such as Newcastle disease virus and avian influenza virus, which can be deadly to chickens. The beneficial role of Src in modulating the immune response remains largely unknown in ducks. Here, we characterized the duck *Src* and found that it contains a 192-base-pair 5′ untranslated region, a 1602-base-pair coding region, and a 2541-base-pair 3′ untranslated region, encoding 533 amino acid residues. Additionally, *duSrc* transcripts were significantly activated in duck tissues infected by Newcastle disease virus compared to controls. The *duSrc* transcripts were notably widespread in all tissues examined, and the expression level was higher in liver, blood, lung, pancreas, and thymus. Moreover, we found the expression levels of IFN-β, NF-κB, IRF3, and *Src* were significantly increased in DEFs after infection with 5′ppp dsRNA, but there was no significant difference before and after treatment in DF1 cells. Furthermore, overexpression of duSrc followed by stimulation with 5′ppp dsRNA led to an elevation of IFN-β levels. The SH3 and PTKc domains of duSrc contributed to promoting the activity of IFN-β and NF-κB in DEFs stimulated by 5′ppp dsRNA.

## 1. Introduction

The Src family of kinases (SFKs) are a class of non-receptor tyrosine kinase, including c-Src, Lck, Hck, Fyn, Blk, Lyn, Fgr, Yes, and Yrk, the nine classical members in humans [1]. The family serves as a critical regulator in regulating immune responses, integrin signaling, motility, and carcinogenesis [2]. Wu et al. found that the Src-family protein tyrosine kinase LCK can be phosphorylated by Asn at Tyr 394 and 505, thus enhancing the intrinsic and extrinsic activity and potency of CD8 T cells against tumors [3]. The Src-family kinase Lyn serves as a crucial regulatory protein in the immune response, triggering pro-inflammatory and inhibitory signaling pathways to modulate immune responses within myeloid cells. Disruptions in Lyn signaling have been linked to the onset of autoimmune conditions [4]. Julie et al. demonstrated Lyn plays a critical role in the innate immune response, particularly in the initial stages of the TLR2 signaling pathway, where it serves as a key molecule triggering the activation of NF-κB [5]. Johnsen IB et al. believed that the involvement of c-Src in antiviral immune responses holds substantial biological importance, as it influences the regulation of both IFN-β and the chemokine IP-10, key mediators that facilitate the stimulation and movement of immune cells in response to viral challenges [6]. In summary, the importance of the Src family kinases in regulating immune responses has been increasingly confirmed.

All members of the Src family kinases have a similar structural arrangement, consisting of modular SH3, SH2, and kinase domains (also known as SH1) [7]. Among these, the SH2 domain is one of the most extensively studied and well-understood protein-interaction modules [8]. It holds a crucial position in signal transduction by binding to phosphotyrosine (pTyr) residues, which are essential for the activation of receptor and non-receptor tyrosine kinases. Research indicates that the SH2 domain of the Fyn protein plays an indispensable role in regulating neuronal migration and cortical organization within the brain [9]. Matthias et al. revealed a novel signaling mechanism of Lck kinase in which tyrosine residue 192nd in the SH2 domain plays a key role in fine-regulating the activation process of T cells [10]. The SH3 domain, on the other hand, exhibits important roles that span numerous cellular processes due to its involved in regulating protein–protein interactions, including immune responses, the cell environment, and protein trafficking [11]. SH3-mediated autoinhibited protein conformations can significantly impact the functioning of signaling proteins that lie upstream of numerous cellular processes. Proteins containing the canonical PxxP motif, which is recognized by SH3 domains, are abundant in proteomes [12]. Lim et al. revealed that both the SH3 and SH2 domains of the Lyn protein actively promote the expression of cytokines regulated by RIG-I through their interaction with the RIG-I receptor and its downstream signaling molecule IPS-1 [13]. The PTKc domain contains key sites, such as Tyr 416 in chickens or Tyr 419 in humans, that are critical for the catalytic activity of c-Src and promote substrate phosphorylation. Many studies have shown that phosphorylation of Tyr419 is enhanced after phosphatase treatment of Tyr530, thereby regulating the phosphorylation of substrates associated with cell migration, adaptation, mitosis, and angiogenesis [14]. In conclusion, the function of the SRC protein is closely related to its unique structural composition, and its internal structure is complex and exquisite. Src contains several key regions, such as the SH3 domain, which specifically binds to the PxxP motif and plays an important role in protein–protein interactions. In addition, Src possesses the SH2 domain, which recognizes and binds to other protein molecules containing tyrosine phosphorylated residues, and this binding is often a trigger point in signal transduction. Finally, the C-terminal of Src contains the tyrosine kinase domain, Tyrkc, which is catalytically active and can promote the phosphorylation of tyrosine, thereby activating a series of biological functions downstream. These domains work together to enable Src to exert its diverse biological effects within the cell [15]. In summary, the domains of the Src family kinases are vital for its activity. Given its significant role in cell growth and proliferation, its activity is tightly regulated by multiple mechanisms to maintain homeostasis.

Ducks serve as natural reservoirs and frequent asymptomatic carriers of RNA viruses such as Newcastle disease virus and avian influenza virus, while chickens are highly susceptible [16,17,18]. Previous researches have indicated that ducks’ defense mechanisms provide better protection against less virulent strains of avian influenza [19]. In duck and avian influenza virus, Newcastle disease virus, or other RNA viruses, after a long symbiosis process, the host immune system and pathogens form a complex interaction network. In this process, the host gradually develops a set of effective defense mechanisms, enhancing the ability to resist disease. Src is a founding member of the SFKs, and acts as a bridge connecting membrane receptors with intracellular signaling mechanisms, thus regulating multiple fundamental cellular processes [20]. The importance of these processes in maintaining cellular equilibrium necessitates precision, and therefore implies a requirement for stringent regulation of the kinase. The c-Src kinase plays a role in preserving normal cellular homeostasis, and several mechanisms meticulously govern its expression and activity levels [21]. The activity of the Src kinase is modulated in response to diverse cellular stimuli through allosteric conformational changes. Therefore, this study aimed to clarify the beneficial role of Src in regulating the specific antiviral immune response of ducks. We conducted molecular cloning and identification of the Src gene in ducks, and analyzed their expression levels before and after 5′ppp dsRNA treatment in vitro. To determine the key domain of Src in the innate immune response against virus in ducks, the effect of three different functional regions of the SRC protein on IFN-β production was evaluated by increasing their expression levels separately.

## 2. Materials and Methods

### 2.1. Animals

Ten healthy 20-day-old Cherry Valley ducks, each weighing 1200 g, were purchased from the Yike Food Group Co., Ltd. (Suqian, China). The ducks were divided into two groups; the treatment groups were infected by NDV and the control groups were infected by Phosphate Buffered Saline (PBS). All inoculations were carried out intraorbitally with a volume of 0.1 mL each. Two days after infection, tissue samples were obtained from post-anesthesia ducks. The ducks were fasted for 12 h before being anesthetized. A quantity of 10% chloral hydrate was prepared for intraperitoneal injection at a ratio of 0.1 mL/10 g. The tissues collected included the heart, liver, spleen, lung, kidney, gizzard, breast muscle, leg muscle, duodenum, brain, pancreas, thymus, bursa of Fabricius, and blood. The tissue samples were promptly placed in a minus 80 degrees environment for preservation, while blood samples were maintained at a temperature of minus 20 degrees for extended storage. To evaluate the expression of *duSrc* mRNA in tissues of Newcastle disease-infected ducks, the total RNA was obtained from duck tissues following the method provided by TRIZOL (TIANGEN, Beijing, China). Subsequently, FastKing gDNA Dispelling RT SuperMix (TIANGEN, Beijing, China) was employed for the synthesis of complementary DNA through reverse transcription, which was then utilized in real-time quantitative PCR assays.

### 2.2. Analysis of Duck Src Gene Cloning and Sequencing

To acquire the CDS sequence of duck *Src*, we designed the primers duSrc-FLAG-F and duSrc-FLAG-R based on the anticipated sequences of duck *Src* (GenBank accession number XM_038166058). Using RT-PCR, we amplified the *duSrc* cDNA from the spleen. Subsequently, to obtain the complete cDNA sequence of *duSrc*, we employed the GeneRacer Kit (L1500-01, Invitrogen, Carlsbad, CA, USA) to perform 5′ and 3′ RACE. The primers are detailed in Table 1. Through the NCBI database, the protein sequences of SRC across various species were identified. Clustal W was used for multiple sequence alignment of SRC proteins from multiple species, and MEGA 6 was used for phylogenetic analysis of SRC amino acid sequences from 17 different species. Furthermore, the conserved regions within the duSrc domains were forecasted by employing the Conserved Domains Database Tools available on the NCBI. 

### 2.3. Cell Culture and Treatment

The duck embryo fibroblasts (DEFs) were propagated in complete Eagle’s Minimum Essential Medium (ATCC, Virginia, VA, USA), enriched with a 10% concentration of fetal bovine serum (Gibco, Grand Island, NY, USA). DF-1 was cultured in DMEM basic (Gibco, Suzhou, China), which was complemented with 10% fetal bovine serum (Gibco, Grand Island, NY, USA). 5′ triphosphate double stranded RNA, abbreviated as 5′ppp-dsRNA, acts as a synthetic ligand specifically designed to interact with the retinoic acid-induced protein designated as RIG-I, which was used in this study as the mimicking stimulation. To investigate the impact of 5′ppp dsRNA on the expression of IFN-β, NF-κb, IRF3, and *duSrc*, DEF and DF-1 cells were cultured in six-well plates until they reached 70–80% confluence. Then, the cells were transfected with 5′ppp dsRNA using Lipofectamine 3000 (Invitrogen, Carlsbad, CA, USA), and the samples were collected after 24 h of treatment. Newcastle disease virus strain (NDV) was the standard virulent strain F48E8, which was presented by Liu Xiufan, Academician of Department of Veterinary Medicine, School of Animal Husbandry and Veterinary Medicine, Yangzhou University. The dose of infection per duck was 10^5^ EID_50_.

### 2.4. Plasmid Construction

Using the ClonExpress II One Step Cloning Kit (Vazyme, Nanjing, China), we successfully constructed the plasmids pcDNA3.1^+^-duSrc, pcDNA3.1^+^-duSrc(SH3), pcDNA3.1^+^-duSrc(SH2), and pcDNA3.1^+^-duSrc(PTKc). These plasmids were then sent to TSINGKE Biological Company for DNA sequencing validation. Additionally, based on the predicted sequence of duck *IFN-β* (GenBank accession no. KM032183), we selected a portion of the promoter region (−167 to +63) to create the duIFN-β promoter. The duIFN-β promoter was cloned into the KpnI and BglII sites of pGL3-Basic to form pGL3-IFN-β. All constructed plasmids underwent DNA sequencing verification by TSINGKE Biological Company. The relevant primers used in this process are listed in Table 1. 

### 2.5. RT-qPCR

Primers were designed using Oligo 7 and subsequently synthesized by Thermo Fisher Scientific (as detailed in Table 1). RT-qPCR assays were employed to monitor alterations in the mRNA levels of target genes. Each reaction mixture in this study had a total volume of 20 μL, comprising 10 μL of ChamQ Blue Universal SYBR qPCR Master Mix (Vazyme, Nanjing, China), 2 μL of forward and reverse primer each, 2 μL of diluted cDNA template, and 4 μL of RNase-free water. The quantitative PCR program is based on the instructions of Vazyme SYBR qPCR Master Mix. β-actin in duck samples served as the internal standard for normalization, and the relative gene expression levels were quantified employing the 2^−ΔΔCt^ method.

### 2.6. Luciferase Reporter Assays and ELISA Analysis

DEF cells were seeded onto 24-well plates. After 24 h, DEFs were transfected with 800 ng of duSrc plasmid, duSrc(SH3) plasmid, duSrc(SH2) plasmid, or duSrc(PTKc) plasmid, together with 1 μg pGL3-IFNβ-Luc and 50 ng pTK-RL. A Luciferase activity was assessed using a Dual-Luciferase Reporter Assay Kit (Promega, Madison, WI, USA) on a multi-functional microplate reader, with activity being normalized to that of the internal control vector, pRL-TK. 

For the ELISA assay, cells were lysed with RIPA Lysis Buffer (Beyotime, Shanghai, China), which was complemented with 1% protease inhibitor cocktail (Beyotime, Shanghai, China), and the supernatants were collected after centrifugation at 12,000 rpm for 10 min at 4 °C and subjected to a duck IFN-β ELISA(YJ373650) (mlbio, Shanghai, China), a duck NF-κB ELISA(YJ203957) (mlbio, Shanghai, China), a duck IRF3 ELISA (YJ803219) (mlbio, Shanghai, China), a chicken IFN-β ELISA(YJ059828) (mlbio, Shanghai, China), a chicken NF-κB ELISA (YJ002789) (mlbio, Shanghai, China), and a chicken IRF3 ELISA(YJ965909) (mlbio, Shanghai, China). The levels of IFN-β, NF-κB, and IRF3 within the sample were measured through a multifunctional microplate reader (Tecan Infinite M200 PRO, Bern, Switzerland). The value was achieved by evaluating the absorbance values of the samples against a pre-established calibration curve. 

## 3. Results

### 3.1. Cloning of duSrc and Sequence Analysis

**Figure 1:**
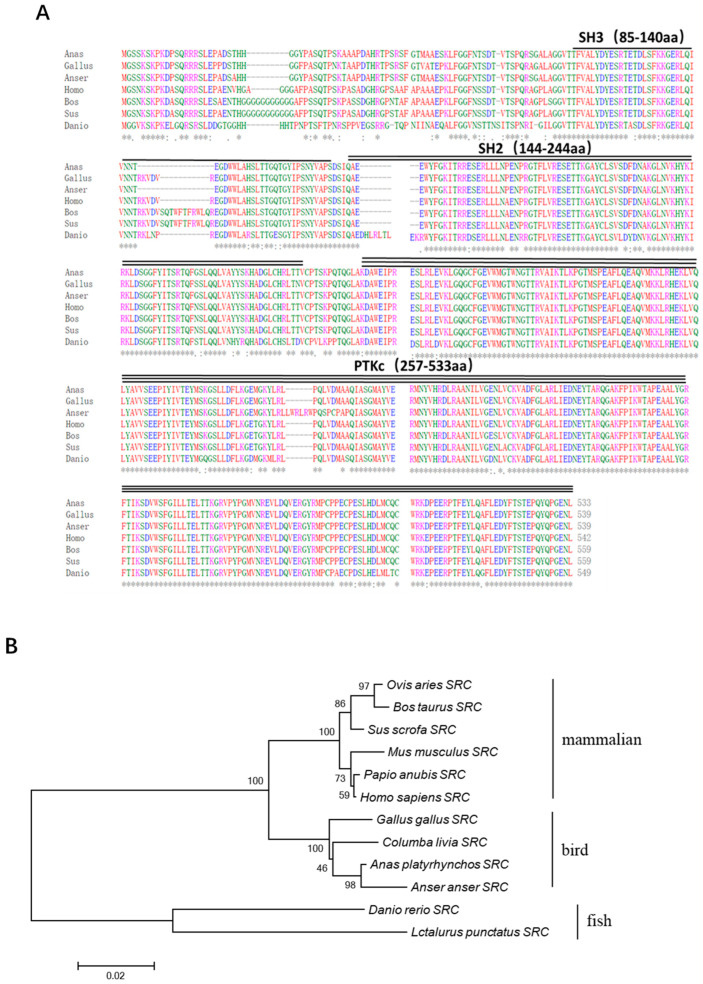
(**A**) The SRC protein sequences of duck (PP987474.1), chicken (NP_990788), goose (XP_047902369), human (NP_938033), cattle (NP_001104274.1), pig (XP_020933628.1), and zebrafish (NP_001003837.2) were performed in multiple alignments. The symbol ‘*’ is used here to identify 100% conserved residues. (**B**) Using version 6 of MEGA software, phylogenetic trees derived from SRC amino acid sequences were constructed via the neighbor-joining method. The protein sequences for each species were sourced from the NCBI GenBank database, which ensured the authority and accuracy of the data. The bar visually shows the percentage of bootstrap values for each branch to facilitate the observation and interpretation of confidence levels in evolutionary relationships.

The complete coding sequence of duck *Src* cDNA was successfully acquired through a combination of RT-PCR and RACE techniques. The results showed that the full length of the duSrc gene consists of three parts: a 192-base-pair 5′ untranslated region (5′UTR), a 1602-base-pair coding region (open reading frame), and a 2541-base-pair 3′ untranslated region (3′UTR), encoding 533 amino acids (GenBank accession number: PP987474.1). Using the Conserved Domain Database tool provided by NCBI, we performed a conserved domain prediction for duSrc. The predicted results revealed that duSrc, as a member of the Src family kinases, has a domain layout that is highly consistent with other members of the family, including the SH3 domain (85–140 aa), SH2 domain (144–244 aa), and PTKc domain (257–533 aa) (Figure 1A).

Utilizing the amino acid sequences derived from bird, mammalian, and fish SRC proteins, a phylogenetic analysis was conducted to elucidate the molecular evolutionary connections among duSRC orthologues across various vertebrate species, as depicted in Figure 1B. DuSRC shared a distant evolutionary relationship with mammal and fish SRC, but had relatively high homology with SRC in poultry, especially geese. The homology of SRC between duck and goose was the highest, at 97.59%. Duck and chicken SRC had the second highest homology, of 96.85%.

### 3.2. Tissue Expression Profile of Src in Ducks Treated with Newcastle Disease Virus

**Figure 2:**
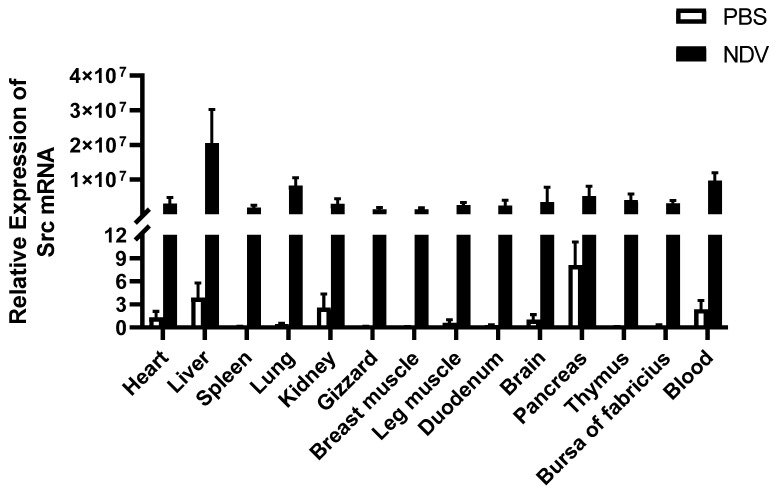
The relative expression levels of the duSrc gene were compared in two groups of Cherry Valley ducks: one group treated with PBS and the other group infected with Newcastle disease virus. The heart, liver, spleen, lung, kidney, gizzard, breast muscle, leg muscle, duodenum, brain, pancreas, thymus, bursa of Fabricius, and blood were tested. β-actin gene expression was used to standardize mRNA levels in each sample. The experimental data are presented as the average of five independent experiments with standard errors.

To investigate the expression patterns of *duSrc* in different tissues of healthy ducks and Newcastle disease virus-infected ducks, we extracted total RNA from different tissues and detected the expression level of *duSrc* transcripts by RT-qPCR. The analysis revealed that *duSrc* was widely expressed in all tissues tested. Compared with the control group, the expression of *duSrc* in tissues of Newcastle disease virus-infected ducks was significantly increased. Among them, *duSrc* transcripts showed the highest expression levels observed in the liver, blood, lung, pancreas, and thymus, with a moderate level in the brain, bursa of Fabricius, heart, kidney, leg muscle, and duodenum, and relatively subdued levels in the spleen, breast muscle and stomach (Figure 2). The results indicated that *duSrc* was significantly activated in the blood, pancreas, thymus, and other immune-related tissues after NDV infection.

### 3.3. Response of Chicken and Duck Embryonic Fibroblasts to 5′ppp dsRNA Stimulation 

**Figure 3:**
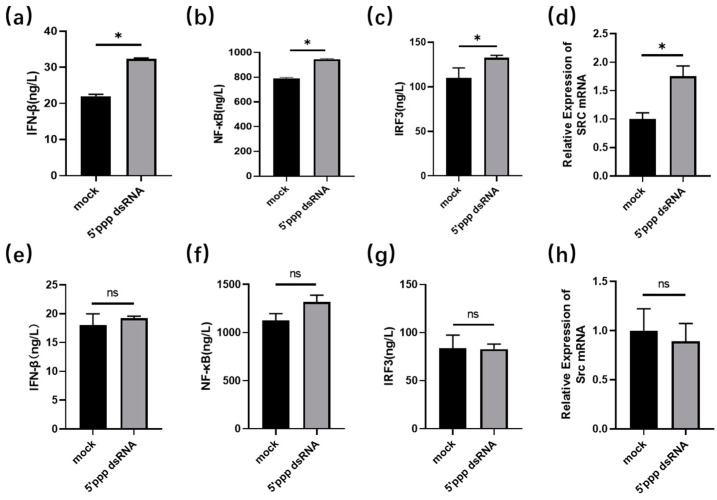
Response of chicken (**e**–**h**) and duck embryonic fibroblasts (**a**–**d**) to 5′ppp dsRNA stimulation. The DEF and DF1 cells were cultured separately in six-well plates until their growth state reached a density of about 70 to 80 percent. Subsequently, Lipofectamine 3000, a highly effective transfection agent, was used to transfer 5′ppp dsRNA into these cells to simulate RNA virus stimulation. Cell precipitates were collected, and proteins and RNA extracted, for detection of the expression level of IFN-β, NF-κB, and IRF3 by ELISA, and detection of the mRNA expression level of *duSrc* by RT-qPCR (*n* = 3). In the statistical analysis, we applied the unpaired two-tailed *t*-test (* *p* < 0.05).

To understand the difference between chickens and ducks facing 5′ppp dsRNA stimulation in vitro, we treated DEF and DF1 cells with 5′ppp dsRNA, respectively, and then examined IFN-β, NF-κB, and IRF3 using ELISA, and the mRNA level of *duSrc* using RT-qPCR. The findings demonstrated the expression levels of IFN-β, NF-κb, IRF3, and *Src* were significantly increased in DEF cells after infection with 5′ppp dsRNA, but there was no significant difference before and after treatment in DF1 (Figure 3). 

### 3.4. The Induction Ability of duSRC and Its Domain to IFN-β

**Figure 4:**
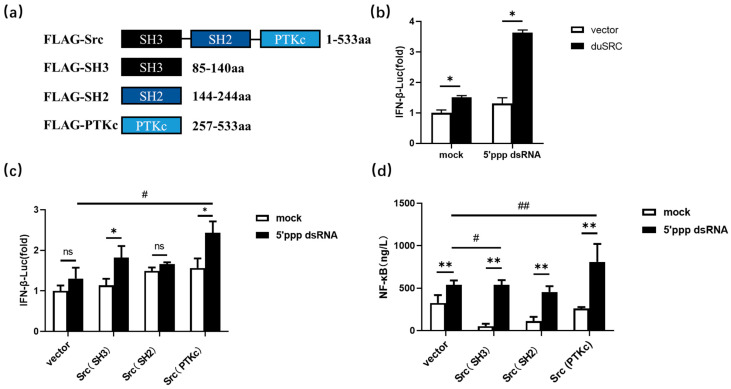
(**a**) Amino acid length and linking order of three domains of DuSrc. We constructed seukaryotic expression vectors of duSrc full-length and three domains to study the role of different duSrc domains in the innate immune antiviral process. Src represents the ORF of duSrc. Src (SH3) represents the SH3 domain of duSrc. Src (SH2) represents the SH2 domain of duSrc. Src (PTKc) represents the PTKc domain of duSrc. (**b**,**c**) Effects of duSrcfull-length and different domains on IFN-β production in duck embryo fibroblasts stimulated by 5′ppp dsRNA. We transfected Src, Src (SH3), Src (SH2), and Src (PTKc) plasmids into DEFs cells with PRL-TK Renilla luciferase plasmids and IFN-β luciferase reporter genes. After 24 h, one group treated the cells with 5′ppp dsRNA for 12 h and the other with saline to compare the effects of different stimulation conditions on IFN-β production. After that, we used the dual-luciferase reporter gene assay to quantify the level of luciferase activity. (**d**) Three different domains of duSrc were transfected into duck embryonic fibroblasts, and after 24 h of transfection, the cells were stimulated with 5′ppp dsRNA (800 ng/well). After 12 h of transfection, the cells were lysed with RIPA, and the proteins were extracted for ELISA experiments. The data are displayed in the form of average values accompanied by standard error measurements, with each set comprising three replicates. Significance was analyzed via one-way ANOVA (* *p* < 0.05, ^#^ *p*< 0.05, ** *p* < 0.01, ^##^ *p* < 0.01, ^ns^ *p* > 0.05).

DuSrc has three conserved domains, namely SH3, SH2, and PTKc, and their connection order is shown in Figure 4a. To investigate the roles of duSrc and its distinct domains, we generated a series of recombinant plasmids expressing various segments of duSrc including Src, Src (SH3), Src (SH2), and Src (PTKc). Subsequently, these recombinant plasmoids were transfected into DEF cells while pcDNA3.1+-FLAG plasmid was used as a negative control group for transfection. The results revealed that an increase in duSrc expression led to the induction of IFN-β following infection with 5’ppp dsRNA. To gain a deeper understanding of the contributions of various duSrc domains to the activation of the duck interferon type I (IFN-I) signaling pathway, a dual-luciferase reporter assay was employed to quantify the activity of the IFN-β gene promoter. The results demonstrated that both the protein tyrosine kinase domain (PTKc) and the SH3 domain of Src significantly enhanced the production of IFN-β when exposed to 5’ppp dsRNA in DEF cells. In addition, overexpression of Src(PTKc) and Src(SH3) notably augmented the generation of NF-κB production in 5′ppp dsRNA-treated cells by ELISA. In general, overexpression of both the PTKc and SH3 domains can partially enhance the antiviral activity of duSrc. Consequently, these domains could play a crucial role within the Src protein, contributing significantly to the duck’s antiviral defense mechanisms.

## 4. Discussion

Innate immunity is an evolutionarily conserved mechanism that differentiates between the self and non-self to safeguard the host from pathogenic intrusion, which includes viruses [22,23]. This system employs various components to recognize pathogenic nucleic acids, and one of the most important receptors is the cytosolic RNA helicase RIG-I [24,25]. Upon viral infection, multiple pattern recognition receptors (PRRs) are triggered to induce the production of type I interferons (IFNs) [26]. Studies have shown that the RIG-I signaling pathway, which recognizes influenza viruses and activates the body’s immune response, activates the production of IFN-β in ducks and exerts an antiviral effect, which is not present in chickens [27]. Double-stranded RNA (dsRNA) molecules with a 5′ triphosphate (5′ppp) end can act as pathogen-associated molecular patterns (PAMPs) that are recognized by RIG-I [28], leading to the production of IFN-Is, which include IFN-α and IFN-β. These cytokines play crucial roles in the defense against viral infections by inducing an antiviral state in infected cells and by modulating the immune system’s response to viral invasion. In this study, DEF and DF1 cells were treated with 5′ppp dsRNA to simulate RNA virus infection in vitro. The results showed that the expression levels of IFN-β, NF-κB, IRF3, and *Src* were significantly increased in DEF cells after infection with 5′ppp dsRNA, but there was no significant difference before and after treatment in DF1. The lack of RIG-I receptors in chickens may be responsible for the difference. When stimulated by 5′ppp dsRNA, chickens cannot produce type I interferons in time to fight viral infection, but ducks can rapidly increase the expression of *Src*, activate NF-κB and IRF3, and eventually produce IFN-β. Evseev et al. demonstrated that in mallards infected with highly pathogenic avian influenza (HPAI), type I interferons and a series of antiviral genes that respond to interferons are rapidly and significantly activated. This process highlights the critical role of the RIG-I signaling pathway in defense mechanisms [16]. Additionally, ducks seem to restrict the duration of this response, especially concerning the expression of pro-inflammatory cytokines. In contrast, chickens, which lack the RIG-I protein, may be at a disadvantage in initiating an early interferon response due to deficiencies in certain modulators of the signaling pathway, leading to increased viral replication and subsequent tissue damage. 

As non-receptor tyrosine kinases, Src-family kinases are very important to organisms, particularly in the regulation of immune responses, integrin signaling, cell motility, and the development of cancer. These kinases influence the health and disease status of the organism by modulating various cellular functions. Johnsen IB et al. confirmed that Src plays a role in the antiviral response triggered by RIG-I. Specifically, they found that Sendai virus (SV), a pathogen detected by RIG-I, leads to phosphorylation and activation of c-Src [6]. Li et al. found that Src plays a crucial role in initiating the activation of the TBK1-IRF3 signaling cascade, thereby enhancing the production of type I interferons. This process involves the stimulation of TBK1 phosphorylation at Tyr179 following viral infection in RAW264.7 macrophage cells [26]. It can be inferred that the tyrosine kinase Src may play an important role in the RIG-I mediated antiviral signaling pathway. In this study, we identified *duSrc* from Cherry Valley ducks. The full length of the duSrc gene consists of three parts: a 192-base-pair 5′ untranslated region (5′UTR), a 1602-base-pair coding region (open reading frame), and a 2541-base-pair 3′ untranslated region (3′UTR), encoding 533 amino acids. Using the Conserved Domain Database tool provided by NCBI, we performed a conserved domain prediction for duSrc. The predicted results revealed that duSrc, as a member of the Src family kinases, has a domain layout that is highly consistent with other members of the family, including the SH3 domain (85–140 aa), SH2 domain (144–244 aa), and PTKc domain (257–533 aa).

In addition, the expression abundance of *Src* in different tissues of healthy Cherry Valley ducks and Newcastle disease virus-infected ducks was investigated for the first time. The results indicated that *duSrc* was widely expressed in all tissues tested before and after virus infection. Consistent with the findings of Corey et al., *Src* is expressed in different tissues of the body [29]. In mammals, the three proteins Src, Fyn, and Yes exist almost everywhere in all types of cells, showing a wide range of distribution properties. However, their congeners Lck, Hck, Fgr, Lyn, and Blk exhibit more specialized expression patterns, focusing more on cells in the hematopoietic system and displaying more limited tissue distribution characteristics. This differential expression reflects their specialized roles in different functional areas of the organism [30]. Compared with the PBS treatment group, the expression of *duSrc* in tissues of Newcastle disease virus-infected ducks was significantly increased, with higher levels in tissues associated with immune function such as the thymus, pancreas, and blood. kang et al. found that Newcastle disease virus caused systemic infection in ducks, including all tested tissues, such as the small intestine, cecal tonsil, brain, lung, bursa of Farsi, thymus, and spleen, with high virus titer and rapid replication in multiple tissues, especially the thymus tissue [31]. In addition, Newcastle disease virus was found to induce significant up-regulation of PRRs, including RIG-I, in lung and thymus tissues of infected ducks, followed by induction of cytokine and chemokine expression [32]. This may explains the significant activation of duSrc in immune organs before and after NDV infection, which implies that duSrc may be involved in antiviral immune response through the RIG-I signaling pathway.

In the current study, we showed a significant increase in *duSrc* mRNA levels following 5′ppp dsRNA infection within duck embryo fibroblast (DEF) cells after 24 h. These findings suggest that duSrc may play a role in mediating an antiviral immune response through the retinoic acid-inducible gene I (RLR) signaling pathway in avian cellular systems. To further demonstrate the role of Src in duck antiviral immune response, we overexpressed Src plasmid in DEF cells and found that it significantly induced IFN-β expression after 5′ppp dsRNA stimulation. As previously mentioned, the antiviral activities of SRC proteins are intimately associated with their different domains. To further investigate the effects of different domains on IFN-β and NF-κB production, we conducted experiments in duck embryonic fibroblasts. By transfecting vectors containing three distinct domains of duSrc, we evaluated the effect of these domains on the dual- luciferase activity of IFN-β and the expression level of NF-κB as measured by enzyme-linked immunosorbent assay (ELISA). Before and after 5′ppp dsRNA transfection, we observed enhanced production of both IFN-β and NF-κB upon the overexpression of the PTKc and SH3 domains. In conclusion, increasing the expression of SH3 and PTKc domains of duSrc in vitro stimulated IFN-β and NF-κB production. NF-κB is a critical mediator of virus-induced IFN-β expression [33,34]. IFN-β, in turn, restricts viral replication and promotes viral clearance by inducing the expression of antiviral genes and modulating virtually all aspects of innate and adaptive immunity [35,36], illustrating that overexpression of the SH3 and PTKc domains plays a crucial role in boosting antiviral responses. Y416 is one of the most important tyrosine residues in Src and located in the kinase domain. The autophosphorylation process of Y416 triggers a significant enhancement in Src kinase activity. This may be the reason why overexpression of PTKc domain can increase the expression of IFN-β and NF-κB. The main function of the SH3 domain is to recognize PxxP motifs [37]. 5′ppp dsRNA stimulation specifically leads to up-regulation of RIG-I expression. The PxxP motif in RIG-I, located on the N-terminal CARDs, recognizes the Src SH3 domain more, leading to increased IFN-β expression. Takuma et al. clarified Src’s distinctive role in the organization of the cytoskeleton within osteoclasts. Both the SH3 and kinase domains played pivotal roles in establishing Src’s functional capabilities. However, the SH2 domain of Src could be functionally replaced by the SH2 domains of either Lyn or Hck [38]. Overall, our findings shed light on the role of Src and its three distinct domains in duck RNA virus recognition, which not only provides new insights into how cells respond to viral invasion, but may also inspire us to design more effective antiviral interventions.

## 5. Conclusions

In conclusion, duSRC shared a distant evolutionary relationship with mammal and fish SRC, but had relatively high homology with SRC in poultry, especially geese. *DuSrc* expression was significantly activated in tissues of Newcastle disease virus-infected ducks. Among them, *duSrc* transcripts were notably elevated in tissues such as the liver, blood, lung, pancreas, and thymus, while they showed relatively low expression in the spleen, breast muscle, and stomach. Compared with DF1 cells, we observed that 5ppp dsRNA stimulation significantly enhanced the expression levels of NF-κB, IRF3, and *Src* in DEF cells. Further studies showed that when duSrc expression was specifically enhanced in duck embryo fibroblasts, the introduction of 5′ppp dsRNA significantly promoted IFN-β production. The PTKc and SH3 domains of duSrc contributed to boosting IFN-β and NF-κB activity in DEFs stimulated by 5′ppp dsRNA. These data might shed light on the defensive mechanisms employed by ducks during viral infections, offering fresh perspectives on their protective immune responses.

## Figures and Tables

**Table 1:** Primers used in this study.

Primer Name	Primer Sequence (5′→3′)	Temperature (℃)	Note
duSrc-Flag-F	ccaagctggctagttaagcttATGGGGAGCAGCAAGAGCA	68	RT-PCR
duSrc-Flag-R	tgctggatatctgcagaattTATAGGTTCTCGCCGGGCTG		
5′ Router	GCCGCTTTGCTGGGTGTCTG	60	5’ RACE-PCR
5′ Inner	CCCCGTGGTGGGTGCTGTCG		
3′ Router	GCAAGGTGCCAAGTTCCCCATC	60	3’ RACE-PCR
3′ Inner	CATCCTGCTGACCGAGCTGACCAC		
duSrc(SH3)-Flag-R	ccaagctggctagttaagcttACCTTCGTGGCCCTCTACGA	55	RT-PCR
duSrc(SH3)-Flag-R	tgctggatatctgcagaattcTGAGGGCGCGACATAGTTACT		
duSrc(SH2)-Flag-F	ccaagctggctagttaagcttCAGGCTGAAGAGTGGTATTTCGG	55	RT-PCR
duSrc(SH2)-Flag-R	tgctggatatctgcagaattcGACGGTGGTCAGACGGTGG		
duSrc(PTKc)-Flag-F	ccaagctggctagttaagcttAAGGATGCCTGGGAAATCCC	55	RT-PCR
duSrc(PTKc)-Flag-R duIFN-β-promoter-F duIFN-β-promoter-R qduSrc-F qduSrc-R qchSrc-F qchSrc-R	tgctggatatctgcagaattcTAGGTTCTCGCCGGGCTG CCCAAGCTTAAGCGATGGGAAAGATGT GGAAGATCTTGTAGGGGCTATGTGGT ATGGGGAGCAGCAAGAGCAAACCCA GGGGTCTTTGGGTTTGC ACTTCTGACACCGTCACGTC ACCAGTCACCTTCCGTGTTG	58 60 60	RT-PCR RT-qPCR RT-qPCR

## Data Availability

The original contributions presented in the study are included in the article, further inquiries can be directed to the corresponding author.
